# Ingrowing thyroglossal duct cyst presenting as a throat mass: A clinical pearl illustrated in two adult patients

**DOI:** 10.1016/j.ijscr.2025.111377

**Published:** 2025-04-26

**Authors:** Dillon D. Joo, John Y. Kim, Justin D. McLarty, Daekeun Joo

**Affiliations:** Department of Head & Neck Surgery, Kaiser Permanente Riverside Medical Center, 10800 Magnolia Avenue, Riverside, CA 92505, United States of America

**Keywords:** Thyroglossal duct cyst, Adult presentation, Intralaryngeal mass, Intralingual cyst, Sistrunk procedure

## Abstract

**Introduction and importance:**

Thyroglossal duct cyst (TGDC) is the most common congenital neck cyst and typically presents as an upper midline neck mass. Rarely, the cyst may present as a pharyngolaryngeal mass by forming posterior to the hyoid without an external palpable component and can present in adults with symptoms that mimic that of oropharyngeal or laryngeal malignancies.

**Case presentation:**

We evaluated two adult male patients for a chief complaint of dysphagia and globus sensation. Both patients had exophytic submucosal masses arising from the vallecula or hypopharynx, raising concern for neoplasm. Incisional biopsy was frustrated by the submucosal location and found to be negative on pathologic examination. However, a diagnosis of intralaryngeal and intralingual TGDC was made radiographically after reviewing the post-biopsy imaging.

**Clinical discussion:**

Eventually both patients underwent the Sistrunk procedure, yielding definitive diagnosis and resolution of their symptoms.

**Conclusion:**

Our cases serve as a reminder that for any cystic mass involving the hyoid bone, presentation in a very specific subset of adult patients may show physical exam findings that would lead to workup of an upper aerodigestive tract malignancy, however TGDC should be considered especially after imaging is thoroughly reviewed.

## Highlights

• Thyroglossal duct cysts can have an upward and ingrowing pattern in adult patients.
• Two patients had symptoms that mimicked those of a throat mass prompting workup for an upper aerodigestive tract malignancy.
• Further workup including computed tomography imaging revealed intralaryngeal and intralingual TGDCs in these patients.

## 1 Introduction

Thyroglossal duct cysts (TGDC) are a type of congenital pathology located anywhere along the thyroid migration track in between the tongue and the inferior neck. These cysts generally occur as a single neck mass anywhere between the foramen cecum and thyroid gland and they form as a result of the development of a fibrous tract that prevents the thyroglossal duct from closing. 70 % of all congenital neck abnormalities are caused by TGDCs [1], and the majority present in the pediatric or adolescent populations. Ingrown TGDCs (intralingual, intralaryngeal and translaryngeal) are even more rare than the classic variant that are seen in the midline neck [2]. We present an unusual case of an ingrown TGDC in two *adult* patients: an intralingual TGDC that presented as a tongue base mass and another in which an intralaryngeal TGDC presented as a hypopharyngeal mass. Both patients originally underwent initial workup to rule out an upper aerodigestive tract malignancy including in-office flexible fiberoptic laryngoscopy and biopsy and even direct laryngoscopy and biopsies in the operating room. However the correct diagnosis was made radiographically once the computed tomography (CT) scans were reviewed, especially in the sagittal plane.

## 2 Methods

This work was completed in line with the SCARE criteria [8].

## 3 Case report

**Fig. 1:**
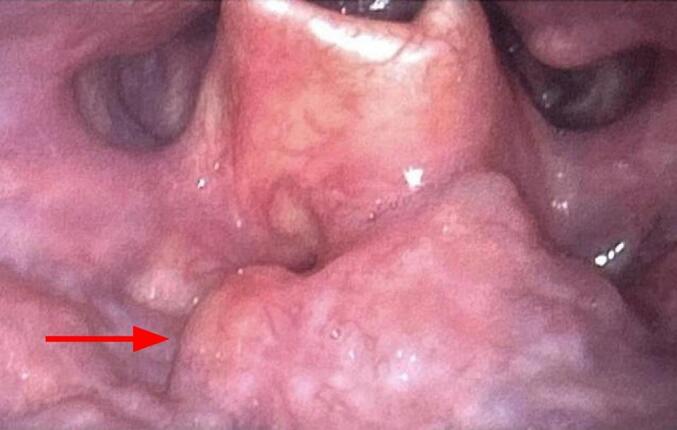
Initial findings on flexible, fiberoptic laryngoscopy (FFL) on Patient 1. Note the exophytic, submucosal vallecular mass.

**Fig. 2:**
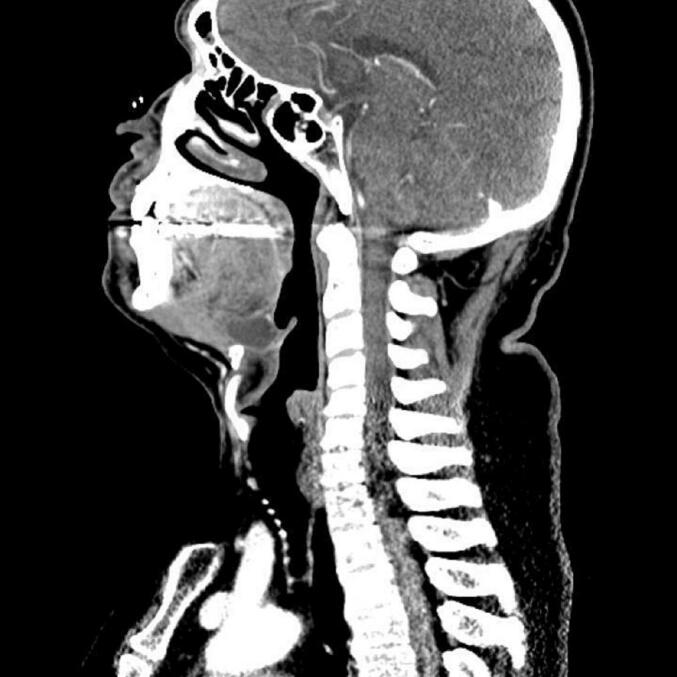
Sagittal, contrast-enhanced computed tomography (CT) image of Patient 1 demonstrates a cystic lesion above the hyoid bone.

**Fig. 3:**
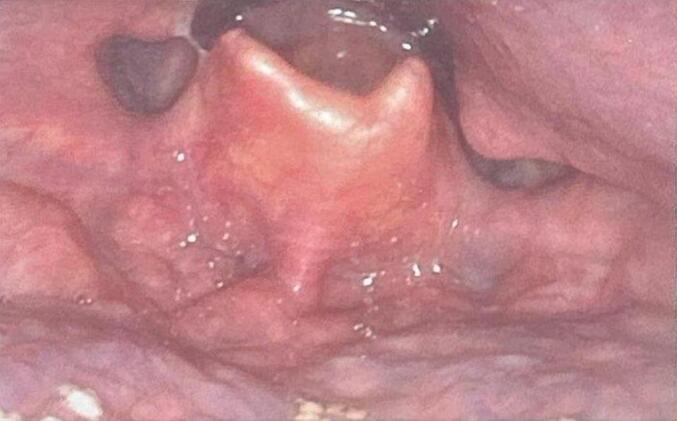
Repeat image of Patient 1 three months post-op, shows resolution of the previous vallecular lesion.

Patient 1: A 62 year-old male presented to the Otolaryngology-Head and Neck clinic with a chief complaint of 4–6 months of dysphagia and persistent left-sided globus sensation. The patient's past medical history is significant for pre-diabetes and gout. He was a non-smoker and denied smoking in the past. A comprehensive Head and Neck exam was performed including flexible, fiberoptic laryngoscopy (FFL). Findings on exam showed an exophytic, submucosal mass arising from the vallecula and left base of tongue (Fig. 1). An attempted in-office biopsy proved negative for malignancy. Thus, further workup including labs and imaging were obtained as well as direct laryngoscopy with deeper biopsies in the operating room. This second biopsy proved to be negative for malignancy as well. Upon review of CT and positron emission tomography (PET) scans, it was determined that the patient's actual diagnosis was an intralingual TGDC. The CT scan showed a 2.3-cm mass in the midline tongue base, suspicious for neoplasm (Fig. 2), and the PET scan did not show any hypermetabolic activity within the oropharynx or hypopharynx. The patient was then taken to the operating room and a standard Sistrunk procedure/excision of TGDC was performed. The final pathology report showed a benign squamous epithelial-lined cyst with adjacent fibrosis. The patient was seen three months later for a postoperative visit and his dysphagia and globus sensation had improved. Repeat FFL showed that the base of tongue mass had resolved. (Fig. 3).

**Fig. 4:**
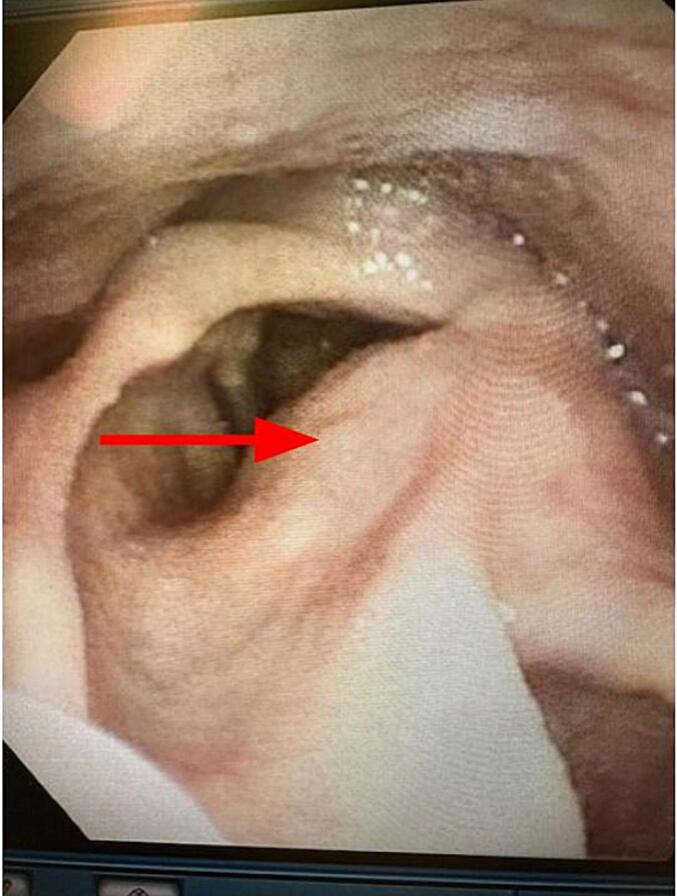
Initial photograph of Patent 2 showing what appears to be a mass effacing the left aryepiglottic fold and piriform sinus, obscuring the glottic opening.

**Fig. 5:**
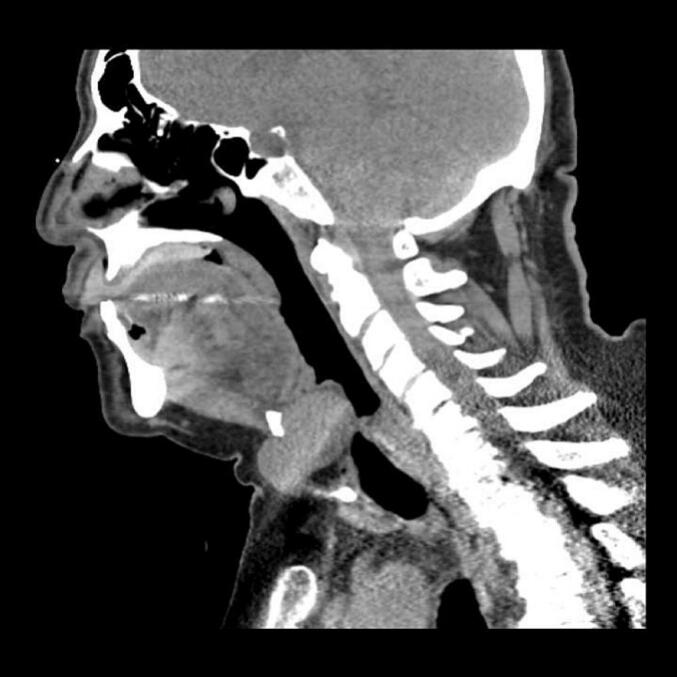
Sagittal, contrast-enhanced computed tomography (CT) image of Patient 2 shows a cystic lesion with upward growth posterior to the hyoid bone.

**Fig. 6:**
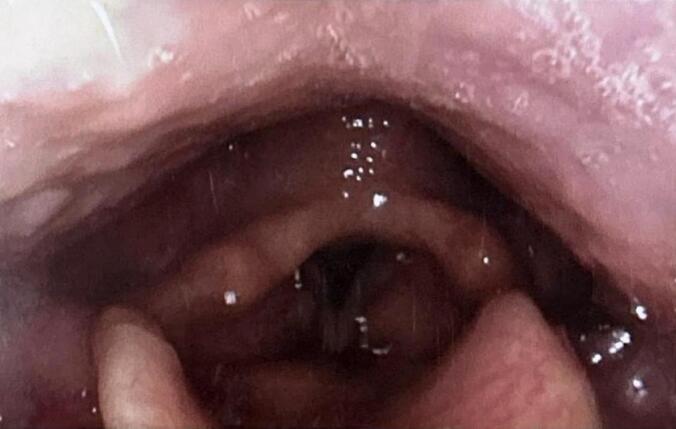
View on FFL of Patient 2 three months post-Sistrunk procedure demonstrates complete resolution of previous left supraglottic lesion and normal view of the glottis.

Patient 2: A second adult male patient presented to the clinic with similar symptoms. He was 69 years old at the time and complained of several months of worsening dysphagia. His PMH was significant for obstructive sleep apnea, type 2 diabetes and hypertension and was a former heavy smoker who had recently quit. On FFL, there was a well-circumscribed, submucosal mass that seemed to be arising from the left aryepiglottic fold (Fig. 4). In-office biopsies were performed with the assumption of a hypopharyngeal malignancy; however these were negative. On closer review of the CT scan that the patient had undergone prior to the initial consultation, the lesion was seen to arise from just below the hyoid bone anteriorly and extended superiorly to efface the larynx above the vocal cords (Fig. 5). It was determined to be an intralaryngeal TGDC. He ultimately underwent a Sistrunk procedure, and intraoperatively he was found to have a 5 cm multi-lobulated, cystic mass located above and below the hyoid bone. Final pathology confirmed the diagnosis of an epithelium-lined cyst associated with thyroid follicles compatible with a TGDC. At two months post-surgery, the patient's dysphagia resolved and repeat FFL showed complete resolution of the hypopharyngeal bulge (Fig. 6).

## 4 Discussion

Thyroglossal duct cysts (TGDC) are one of the most common congenital neck masses in children and usually present as a midline neck mass that occurs anywhere between the foramen cecum of the tongue and the suprasternal notch, but most often in the anterior neck, inferior to the hyoid bone and closely related to the thyrohyoid membrane. However about 8 % can present above the hyoid bone and approximately 1–2 % at the base of tongue [1]. Although commonly found in the pediatric population, TGDCs are also found in adults with a reported prevalence of approximately 7 % [3]. Within the adult group, there is an even more exclusive subset of patients that present with a TGDC originating above the hyoid bone, without a palpable neck mass, extending into the oropharynx or larynx with physical exam findings of a visible mass seen on FFL. These patients are sometimes misdiagnosed and have been subject to workup to rule out malignancy which can include in-office biopsies and even direct laryngoscopy with biopsies in the operating room.

It is well-established that the position of the thyroglossal duct lies anterior to the body of the hyoid bone [4]. This would explain why most TGDCs present as midline anterior neck masses. There have been reported cases of intralaryngeal TGDCs originating from a posteriorly located thyroglossal duct [5]. Zhang et al. in a study of 250 patients with TGDCs, 7 patients (2.8 %) showed a pattern of ingrowth in which the cyst arose from a thyroglossal duct either posterior to the hyoid bone or above the hyoid bone within the base of tongue [6]. A lesion located posterior to the hyoid bone would grow into the preepiglottic space due to obstruction by the hyoid bone and eventually into the supraglottic larynx. With enough time these lesions can expand the area of the aryepiglottic fold and false vocal folds enough to appear as a marginal tumor on FFL. These patients often present with months of globus sensation, dysphagia and even hoarseness [7] all of which mimic the history of patients with upper aerodigestive tract malignancies. Similarly lesions that originate close to the foramen cecum above the hyoid bone will tend to grow upward and present as an intralingual cyst. With enough upward growth, these can protrude into the vallecula or base of tongue which is the precise location of many oropharyngeal squamous cell carcinomas.

Both patients in our small series were older male patients that presented to our clinic with a history of persistent globus sensation and/or dysphagia and hoarseness. Given their age and gender with one of the patients having a prior smoking history, the findings on FFL prompted a workup to rule out malignancy. Because of this urgency, both patients underwent attempted in-office biopsies through a video laryngoscope with a working port. CT and PET scans were ordered after the initial consultation and reviewed post-biopsy. On closer review of the CT scans, especially in the sagittal plane, it was determined that in both cases a thyroglossal duct cyst had originated above or posterior to the hyoid bone causing inward or upward growth enough to show physical exam findings that mimicked a mass within the oropharynx or hypopharynx. Both patients underwent successful Sistrunk procedures and their symptoms resolved within 3 months of recovery.

## 5 Conclusion

Although not as common as in children, TGDCs are also seen in some adult patients. A small subset of these patients may present with findings similar to those with upper aerodigestive tract malignancies. Initial workup may include FFL and biopsies; however a thorough review of the imaging should follow which will often lead to the correct diagnosis. Thus patients with a submucosal mass in the oropharynx or larynx with characteristic radiographic findings on CT, consideration should be made for the possibility of an intralaryngeal or intralingual TGDC.

## Consent

Written informed consent was obtained from the patient for publication of this case report and accompanying images. A copy of the written consent is available for review by the Editor-in-Chief of this journal on request.

## Ethical approval

Kaiser Permanente Institutional Review Board review and approval was waived for this case report because the activity does not meet the regulatory definition of research as defined by 45 CFR 46.102 (l) “A systematic investigation, including research development, testing and evaluation, designed to develop or contribute to generalizable knowledge”. This determination was as per Standard Operating Procedure (SOP) 013 “Determining the Need for IRB Review and Exemption of Human Subject Research.”

## Funding

This research did not receive any specific grant from funding agencies in the public, commercial or not-for-profit sectors.

## Author contribution

Daekeun Joo, MD, FACS – Project Administration, Reviewing & Editing

Dillon Joo – Original Draft, Writing, Reviewing & Editing

Justin D. McLarty, MD – Reviewing & Editing, Writing, Supervision

John Y. Kim, MD - Supervision.

## Guarantor

Daekeun Joo.

## Research registration number

Not Applicable.

## Declaration of competing interest

The authors declare that they have no known competing financial interests or personal relationships that could have appeared to influence the work reported in this paper.
